# SARS-CoV-2 detection among international air travellers to Ghana during mandatory quarantine

**DOI:** 10.4314/gmj.v55i2s.7

**Published:** 2021-06

**Authors:** Bright Adu, Joseph K H Bonney, John K Odoom, Evelyn Y A Bonney, Evangeline Obodai, Ivy A Asante, James Aboagye, Mildred A Adusei-Poku, Lawrence Lartey, Ernest K Asiedu, William K Ampofo, George B Kyei

**Affiliations:** 1 Immunology Department. Noguchi Memorial Institute for Medical Research, College of Health Sciences, University of Ghana. Off Akilagpa Sawyerr Road. PO Box LG 581, Legon, Accra, Ghana; 2 Virology Department. Noguchi Memorial Institute for Medical Research, College of Health Sciences, University of Ghana. Off Akilagpa Sawyerr Road. PO Box LG 581, Legon, Accra, Ghana; 3 Department of Microbiology, University of Ghana Medical School. College of Health Sciences, University of Ghana. PO Box GP 4236, Accra, Ghana; 4 Disease Surveillance Department, Ghana Health Service, Private Mail Bag, Ministries, Accra. Ghana; 5 National Quality Management Unit, Ministry of Health, P.O.Box M 44, Ministries, Accra, Ghana; 6 Department of Medicine, Washington University School of Medicine, 660 S. Euclid Avenue, St. Louis, MO, USA

**Keywords:** COVID-19, SARS-CoV-2, mandatory quarantine, Ghana, international travellers, testing

## Abstract

**Objectives:**

To determine the prevalence of SARS-CoV-2 detection among international travellers to Ghana during mandatory quarantine.

**Design:**

A retrospective cross-sectional study.

**Setting:**

Air travellers to Ghana on 21^st^ and 22^nd^ March 2020.

**Participants:**

On 21^st^ and 22^nd^ March 2020, a total of 1,030 returning international travellers were mandatorily quarantined in 15 different hotels in Accra and tested for SARS-CoV-2. All of these persons were included in the study.

**Main outcome measure:**

Positivity for SARS-CoV-2 by polymerase chain reaction.

**Results:**

The initial testing at the beginning of quarantine found 79 (7.7%) individuals to be positive for SARS-CoV-2. In the exit screening after 12 to 13 days of quarantine, it was discovered that 26 of those who tested negative for SARS-CoV-2 in the initial screening subsequently tested positive.

**Conclusions:**

Ghana likely averted an early community spread of COVID-19 through the proactive approach to quarantine international travellers during the early phase of the pandemic.

**Funding:**

None

## Introduction

The Coronavirus Disease 2019 (COVID-19) caused by SARS-CoV-2 has now been reported in nearly every country worldwide and has undoubtedly become the single most important global health challenge.^1^ What was merely described initially as pneumonia of unknown aetiology with typical symptoms now poses a significant threat to global health.^2^ COVID-19 caused several governments to invoke strict local and international travel restrictions and mandatory lockdowns at unprecedented proportions.^3^,^4^ With no approved standard treatment or vaccine during the initial phase of the pandemic, the major control strategy currently against COVID-19 was travel restrictions, physical distancing and identification and isolation of infected individuals through mass screening of high-risk populations.^1^

It is agreeable that international travel was the main means of COVID-19 spread between countries.^4^ As of March 15, 2020, Ghana had recorded six confirmed imported cases and no local transmission.^5^ In line with the global strategy to minimize the importation of COVID-19 cases to less affected countries, the government of Ghana issued a directive on March 15, 2020 to prohibit travellers who have visited countries with 200 or more reported cases of COVID-19 within the last 14 days, before the issuance of the directive, from entering Ghana.^6^ Travellers from 24 countries were affected by the directive, which was to take effect from 17^th^ March 2020. However, Ghanaian citizens and other nationals with Ghana residence permit would be allowed to travel into the country but be subjected to a 14-day mandatory self or government established quarantine. The objective of this study was to describe the COVID-19 results for the travellers who were mandatorily quarantined at a crucial period in Ghana's response and discuss the implications for this and future pandemics.

## Methods

The study examined 1,030 travellers who were quarantined at various locations in Accra. Nasopharyngeal samples were taken from all travellers, transported in viral transport medium and processed at the Noguchi Memorial Institute for Medical Research Virology laboratories. The demographic information was extracted from the Ghana Health Service case investigation forms accompanying the samples. Briefly, after ribonucleic acid (RNA) extraction, samples were subjected to Real-Time Reverse Transcription-Polymerase Chain Reaction (rRT-PCR) using primers that target the SARS-CoV-2 nucleocapsid (N), the open reading frame 1ab (ORF 1ab) and/or the envelope (E) genes. A positive or negative PCR was interpreted according to the kits manufacturers' (Da An Gene, MiRXES and TIBMOLBIOL) interpretation guidelines. A cycle threshold (Ct) value of 40 or above was considered a negative.

## Results and Discussion

The government announced the closure of the Ghanaian borders on 20^th^ March, to take effect from 23^rd^ March 2020. However, on 21^st^ and 22^nd^ March 2020, a total of 1,030 returning international travellers were mandatorily quarantined by the government in 15 different hotels in Accra. Nationality information was available for 885 travellers, of which the majority (88.1%, 780) were identified as Ghanaians while the remaining 105 (11.9%) were of other nationalities. The 819 individuals whose travel history was available were travellers returning from the United Kingdom (41.0%, 336), Nigeria (21.1%, 173), other African countries (21.2%, 174), other non-African countries (12.8%, 105) and USA (3.8%, 31). Out of the 1030 travellers, 39.0% were female. At the beginning of the quarantine period, all the travellers were screened using reverse transcription real-time polymerase chain reaction (RT-PCR) for SARS-CoV-2. A total of 79 (7.7%) individuals were positive, while the remaining 951 were negative. Of those who tested positive, 31 (39.2%) arrived on 21^st^ March 2020, and 48 (60.8%) arrived on 22^nd^ March 2020. Of the positive cases, the majority 55(69.2%) were travellers arriving on a single flight.

These were quite important realizations because had there been no mandatory quarantine and testing, each of these infected individuals would have potentially resulted in several new community infections that would have added to the spread of COVID-19 in Ghana. It was more revealing to note that after 12 to 13 days of quarantine, 26 of those who tested negative for SARS-CoV-2 in the initial screening tested positive. This brought the total number of positive cases to 105, representing 10.2% of the quarantined travellers within the period.^5^ Government decision to mandatorily quarantine returning international travellers can be a commendable initiative, especially given the high reproductive number of SARS-CoV-2.^7^ The contribution of the 105 positive cases to the spread of COVID-19 in Ghana would have been enormous.

Although this strategy may have averted several potential COVID-19 cases, the implications of these findings deserve further deliberations. It is conceivable that COVID-19 may not be the last pandemic the world would have to deal with, so we highlight the most critical questions and draw lessons that could help in such times. Firstly, how reliable are the ports of entry screening based on body temperature in identifying COVID-19 cases? Indeed, this question has also been raised by others.^8^ The measurement of fever as a means of identifying infected individuals at ports of entry into countries became more widespread during the 2013–2016 outbreak of the Ebola virus disease in West Africa.^9^,^10^ However, for a disease like COVID-19, where most cases may remain asymptomatic,^8^,^11^,^12^ screening for infected individuals by temperature measurement alone may not be the most effective approach. This is because all the 1030 travellers who were quarantined in Accra had been initially screened at the airport for fever and were all found to be non-febrile. Thus, based on temperature screening alone, the infected individuals would have all ended up in the population to increase the spread of COVID-19. The limitations of the temperature-based screening method become even critical for travellers who may be concealing fever through antipyretic medication or are in the incubation phase of the infection. One can only wonder exactly how many COVID-19 positive cases had been missed by conventional body temperature screening methods at the ports of entry in Ghana (or other countries) before the mandatory testing of returning travellers and how these have contributed to the current pandemic status of the disease.

Secondly, should there be two-time mandatory testing before a COVID-19 negative status is assigned? It is possible that the 26 individuals who tested negative initially but positive later were in the incubation phase of the infection and the viral load was not yet within the level of detection of the RT-PCR. Two-time testing brings along the cost and logistic implications; however, such a thorough screening strategy may rather be more cost-effective in the long run since the Impact on disease containment or control could be substantial.

Finally, on the global outlook, should more countries have instituted travel and border restrictions much earlier than was done? While the reasons for the relatively late imposition of travel restrictions by various countries, including Ghana, maybe well-meaning and justified, one cannot help but imagine what the situation would have been had those restrictions been instituted much sooner. In their article, Wells and colleagues showed that there was a >95% daily risk of exporting a COVID-19 case from mainland China through international travel by mid-January 2020.^4^ The same study estimated that by mid-February, close to 800 COVID-19 cases would have been exported without travel restrictions imposed by the Chinese government. Perhaps a more aggressive approach to instituting travel restrictions and border control would have slowed down the spread considerably or limited it to smaller geographical regions without reaching pandemic status.

## Conclusion

Ghana likely averted an early community spread of COVID-19 through the proactive approach to quarantine international travellers. However long the COVID-19 pandemic lasts, or widespread its severe Impact would reach, there are several lessons the Ghana Health Service and all key stakeholders of global health security could take from this.
